# Risk Factors for Malformations and Impact on Reproductive Performance and Mortality Rates of Schmallenberg Virus in Sheep Flocks in the Netherlands

**DOI:** 10.1371/journal.pone.0100135

**Published:** 2014-06-17

**Authors:** Saskia Luttikholt, Anouk Veldhuis, René van den Brom, Lammert Moll, Karianne Lievaart-Peterson, Klaas Peperkamp, Gerdien van Schaik, Piet Vellema

**Affiliations:** 1 Department of Small Ruminant Health, GD Animal Health, Deventer, The Netherlands; 2 Department of Epidemiology, GD Animal Health, Deventer, The Netherlands; 3 Department of Pathology, GD Animal Health, Deventer, The Netherlands; The Pirbright Institute, United Kingdom

## Abstract

In Northwestern Europe, an epizootic outbreak of congenital malformations in newborn lambs due to infection with Schmallenberg virus (SBV) started at the end of 2011. The objectives of this study were to describe clinical symptoms of SBV infection, the effect of infection on mortality rates, and reproductive performance in sheep, as well as to identify and quantify flock level risk factors for SBV infections resulting in malformations in newborn lambs. A case-control study design was used, with 93 case flocks that had notified malformed lambs and 84 control flocks with no such lambs. Overall animal seroprevalence in case flocks was estimated at 82.0% (95% CI: 74.3–87.8), and was not significantly different from the prevalence in control flocks being 76.4% (95% CI: 67.2–83.6). The percentages of stillborn lambs or lambs that died before weaning, repeat breeders, and lambs with abnormal suckling behaviour were significantly higher in case flocks compared to control flocks. However, effect of SBV infection on mortality rates and reproductive performance seemed to be limited. Multivariable analysis showed that sheep flocks with an early start of the mating season, i.e. before August 2011 (OR = 33.1; 95% CI: 10.0–109.8) and in August 2011 (OR = 8.2; 95% CI: 2.7–24.6) had increased odds of malformations in newborn lambs caused by SBV compared to sheep flocks with a start of the mating season in October 2011. Other flock-level risk factors for malformations in newborn lambs were purchase of silage (OR 5.0; 95% CI: 1.7–15.0) and flocks with one or more dogs (OR = 3.3; 95% CI: 1.3–8.3). Delaying mating until October could be a potential preventive measure for naïve animals to reduce SBV induced losses. As duration of immunity after infection with SBV is expected to last for several years, future SBV induced congenital malformations are mainly expected in offspring of early mated seronegative animals.

## Introduction

During summer and fall of 2011, dairy cows displayed fever, decreased milk production and diarrhoea especially in the eastern regions of the Netherlands [1] and in North-Rhine Westphalia [2]. Bovine viral diarrhoea virus, bovine herpes virus-1, bluetongue virus, foot-and-mouth-disease virus, epizootic haemorrhagic disease virus, Rift Valley fever virus and bovine ephermeral fever virus were excluded as possible infectious etiologies [1], [2]. On November 18^th^ 2011, the Friedrich Loeffler Institute (FLI), Insel Riems, Germany identified a novel orthobunyavirus, provisionally named Schmallenberg virus (SBV), using virus isolation and metagenomic analysis of serum samples from diseased dairy cattle in a farm near the German town Schmallenberg [2]. Shortly after, an epizootic outbreak of congenital malformations, featuring an arthrogryposis hydranencephaly syndrome, in newborn lambs, kids and calves associated with SBV started in Northwestern Europe [3], [4]. In December 2011, brain tissue samples from Dutch malformed lambs tested positive in the real-time quantitative reverse transcription PCR (RT-qPCR) provided by FLI [4]. This epizootic outbreak of congenital malformations in ruminants is the first orthobunyaviral emerging disease reported in Europe.

In areas with reporting of congenital malformations due to SBV in France, a within-flock seroprevalence of 30% was found in sheep in winter 2011–2012 [5]. Between clinically affected and not-clinically affected flocks no significant differences in seroprevalences were found [5]. A seroprevalence of 89.0% was found in sheep samples sampled between November 2011 and March 2012 in the Netherlands [6]. Preliminary findings in the Netherlands suggest no clinical signs in ewes, except for dystocia, and mild to severe congenital malformations in newborn lambs in affected flocks [4]. In a field study in SBV affected flocks in Belgium, dystocia and abortion were reported as the main clinical signs in adult sheep, in lambs stillbirth and congenital malformations were regularly reported [7], [8]. After experimental infection of 30 sheep in Germany and Denmark, no clinical symptoms were reported, except for one sheep which had diarrhoea for four days, and nasal discharge in two sheep [9].

The objectives of this study were to describe clinical symptoms of SBV infection, the effects of infection on mortality rates, and reproductive performance, as well as, to identify and quantify risk factors for malformations in newborn lambs caused by SBV in Dutch sheep flocks.

## Materials and Methods

### Selection of Flocks

A case-control study design was used. A sample size of 100 case flocks and 100 control flocks was determined in order to detect a risk factor exposure odds ratio (OR) of 2–3, with 95% confidence and 80% power [10]. Out of all sheep flocks that had notified malformations in newborn lambs from the first notification on November 25^th^ 2011 until April 30^th^ 2012 (n = 349), 115 flocks were classified as candidate case flock. Inclusion criteria were (i) birth of malformed lambs caused by SBV based on post-mortem examination at GD Animal Health (GD), and (ii) a minimal flock size of 50 breeding ewes. If two flocks were located in the same four-digit postal code area, one of the flocks was randomly selected. The first 100 flock owners that agreed to participate were included. After the start of data collection, seven case flock owners decided to withdraw from the study because of various reasons. In total, 93 case flocks met the inclusion criteria and agreed to participate. Control flocks were invited to participate through a publication in a professional magazine for sheep owners, by e-mail, and via the GD website. The candidate control flocks had to meet the following inclusion criteria: (i) no malformations in newborn lambs were observed between November 25^th^ 2011 and end of the notification period at the begin of July 2012, and (ii) a minimal flock size of 50 breeding ewes. If two flocks were located in the same four-digit postal code area, one of the flocks was randomly selected. In total, 84 control flock owners that applied to participate met the inclusion criteria.

### Data Collection and Diagnostic Procedures

Five employees of the Department of Small Ruminant Health interviewed flock owners. The questionnaire was pretested on one sheep flock and interpretation of questions and results were discussed with the five interviewers. Between June and October 2012, all case and control flock owners were visited once, and were interviewed about general farm characteristics, insect and parasite control measures, vaccination strategies, sheep health management, the lambing season and malformations in lambs.

To determine the SBV status of a flock, serum samples from ten sheep that had lambed at least once, were collected. Sample size assumptions were a flock seroprevalence of at least 50% with 95% confidence, given an average flocks size of 135 ewes on professional sheep farms (defined as flocks with more than 31 sheep) in the Netherlands [10]. Seven of 177 flocks owners were not willing to submit ten serum samples. To estimate within-flock seroprevalences, 67 flocks were asked to collect 100 serum samples from ewes that had lambed at least once, assuming a within-flock seroprevalence of 50% with 95% confidence and 5% error [10]. Thirty-seven of 67 flocks owners were not able to collect additional serum samples for various reasons. All serum samples were collected before October 30^st^ 2012.

Samples were tested for presence of SBV antibodies using an in-house indirect whole virus ELISA with a relative sensitivity of 98.8% (95% CI: 93.3–99.8) and a relative specificity of 98.8% (95% CI: 97.5–99.6) [11]. Test results were defined as positive, negative or non-specific. Test results were considered to be non-specific when a serum sample responded to the control antigen without viral antigen, irrespective of the gross optical density of the sample.

Participating flock owners were requested to submit all malformed newborn or stillborn lambs for post mortem examination between December 20^th^ and the beginning of July 2012. Submitted lambs were also tested for presence of SBV in brain tissue using the RT-qPCR [2].

This field study was carried out on private flocks, and for collection of serum samples participating sheep flock owners did give permission. Blood samples were collected by the local veterinary practitioner from the jugular vein, using one 10 mL vacuum blood collection tube per animal, to determine the SBV disease status of the flock. For that reason, no specific approval was necessary. Flock owners and veterinarians participated on a voluntary basis and were informed in writing before the start of the study. They also received the results of serological testing and study results. The institutional review board of GD approved the study before it started and reviewed the results.

### Data Analysis

Statistical analyses were performed using STATA/SE version 12.1 software [12]. Overall animal level apparent seroprevalence, and within-flock seroprevalence were estimated using an intercept-only logistic regression model (logit) adapted to the survey design characteristics of the dataset (svyset and svy prefix). By this method, both clustering of animals (or test results) within flocks and sampling weights are taken into account in the estimation procedure. Including sampling weights is necessary as samples from a large flock should receive a higher weight in the overall seroprevalence estimates than samples from smaller flocks, as a fixed (non-proportional) sample size was applied to all flocks. Sampling weights were calculated as the inverse of the sampling probability of an animal, which was calculated as the proportion of sampled animals per flock in relation to flock size. The sampling probability of flocks was considered to be equal across flocks. True animal seroprevalences based on imperfect performance of serological tests were calculated according to Rogan [13], using the estimated apparent seroprevalences. If flock owners submitted more than 100 samples, only 100 samples were randomly selected.

Multivariable logistic regression analyses (logit) were conducted at flock level to describe the relationship between potential risk factors and birth of malformed lambs in 2011 and 2012. All variables obtained from the questionnaire and other data sources were subjected to univariable analysis prior to each multivariable analysis. Only variables showing a *p*-value of less than 0.25 were included in a multivariable model. Final multivariable models were obtained by a backward selection procedure, removing step by step each variable with a *p*-value greater than 0.10. During the selection procedure, confounding of variables was monitored by change in coefficient values. If the relative change exceeded 25% or more, or by 0.1 when the value of the coefficient was between −0.4 and 0.4, the removed variable was considered a potential confounder and re-entered in the model. Multivariable models with the highest R^2^-value are shown. In the final model, all biologically plausible two-way interactions were tested. Goodness-of-fit of logistic models was assessed using Pearson’s goodness of fit test (estat gof). Effects of SBV infection on reproductive performance and mortality rates were tested with a Pearson’s chi-squared test or a *t*-test to compare means of variables with a normal distribution.

## Results

### Participating Flocks

**Figure 1:**
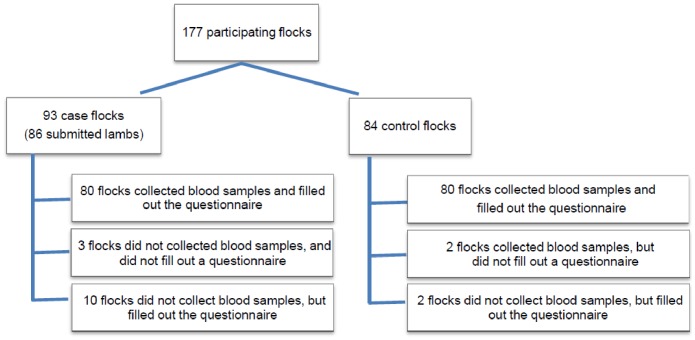
Overview of 177 participating sheep flocks in SBV study in the Netherlands.

**Figure 2:**
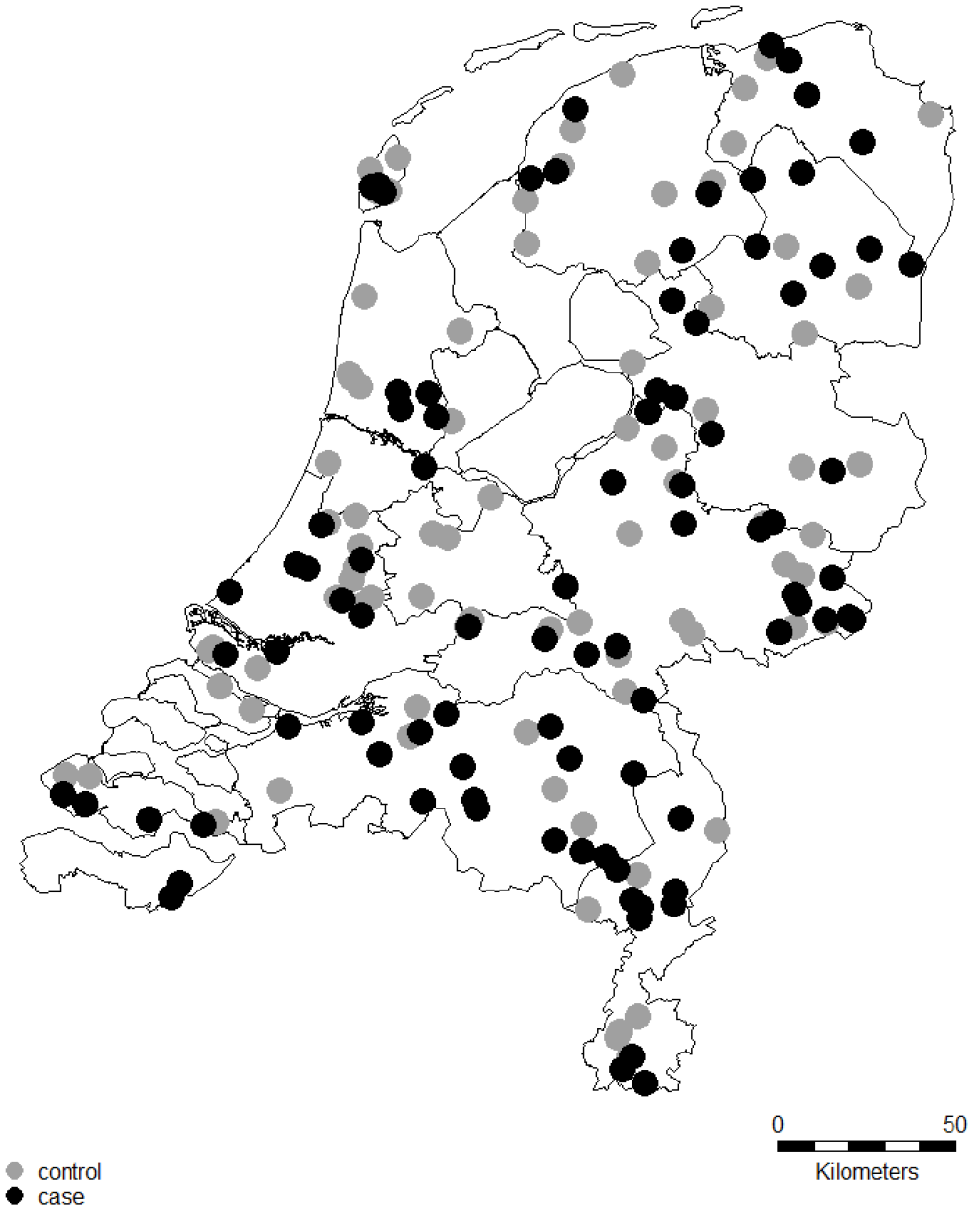
Location of SBV case (black dots) and control (grey dots) Dutch sheep flocks.

From the 177 participating flocks, serum samples from 162 flocks were submitted to the GD (Figure 1). A questionnaire was completed for 172 flocks. Due to incomplete administration of flock owners, information about the moment of birth of malformed lambs, clinical symptoms in ewes and lambs, and effect on reproductive performance and mortality rates were not for all flocks available. From the 93 case flocks, 86 submitted malformed or stillborn lambs for post-mortem examination. Sixty-two flocks were located in the north of the Netherlands, 66 in the central region, and 49 in the south (Figure 2).

### Within-flock Seroprevalences

In total, serum samples from 162 out of 177 flocks were collected from January 5^th^ 2012 to October 30^th^ 2012. Only samples with a negative or positive outcome were used for seroprevalence estimations, excluding 62 samples with non-specific results. A total of 4,227 samples from 80 case and 82 control flocks could be used for further analysis. All 162 flocks had at least one seropositive animal. True and apparent seroprevalences were almost equal due to high test sensitivity and specificity, therefore only apparent seroprevalences are shown. An overall animal-level seroprevalence in 4,277 animals from 162 flocks was estimated at 80.2% (95% CI: 74.4–85.0). Overall animal seroprevalence in 2,066 animals from 80 case flocks was estimated at 82.0% (95% CI: 74.3–87.8), and was not significantly different from the seroprevalence in 2,162 animals from 82 control flocks (76.4%, 95% CI: 67.2–83.6). Within-flock seroprevalence ranged from 15% to 100%, based on case and control flocks that submitted at least fifty samples. The proportion of positive samples in case flocks (76.9%, median = 77.5) was not different from the proportion of positive samples in control flocks (85.0%, median = 94.0) (*p = 0.067,* Pearson’s chi-squared test). In total, 47 flocks submitted samples from both ewes with and without malformed offspring (n = 685 samples). Estimated seroprevalence in ewes that gave birth to clinically normal lambs (n = 475) was 75.7% (95% CI: 58.4–87.3) and did not significantly differ from the seroprevalence in ewes that gave birth to one or more malformed lambs (n = 210), 88.3% (95% CI: 79.1–93.8) (*p = 0.13*).

### Pathological Changes and Clinical Symptoms

In total, 433 malformed lambs from 86 flocks were submitted for post mortem examination, of which 409 were tested for SBV, and of which 34.2% tested positive using RT-qPCR. None of the lambs submitted between March 19^th^ 2012 and May 8^th^ 2012 was SBV RT-qPCR positive. Between May 8^th^ and the end of the notification period at the beginning of July 2012, no lambs were submitted from participating flocks in this study. Arthrogryposis of the fore limbs (74.1%), arthrogryposis of the hind limbs (68.1%), scoliosis (42.0%) and microcephaly (38.1%) were the most observed gross findings (Table 1). Hypoplasia of various regions of the central nervous system appeared as another important finding, reflecting the neurotropic nature of SBV and corresponding with the axial and appendicular skeletal changes through the neuromuscular axis.

**Table 1:** Macroscopic findings observed in submitted necropsied lambs (n = 433) from 86 Dutch sheep flocks with a suspected SBV-infection.

Malformation	Number of lambs	Percentage
*External skeleton*		
Arthogryposis fore limbs	321	74.1%
Arthogryposis hind limbs	295	68.1%
Scoliosis	182	42.0%
Microcephaly	156	38.1%
Torticollis	128	29.6%
Kyphosis	123	28.4%
Brachygnathia inferior	49	11.3%
Lordosis	15	3.5%
*Central nervous system*		
Hypoplasia cerebellum	297	68.6%
Hypoplasia spinal cord	197	45.5%
Hypoplasia cerebrum	161	37.2%
Hydranencephaly	70	16.2%
Hypoplasia brainstem	30	6.9%

**Figure 3:**
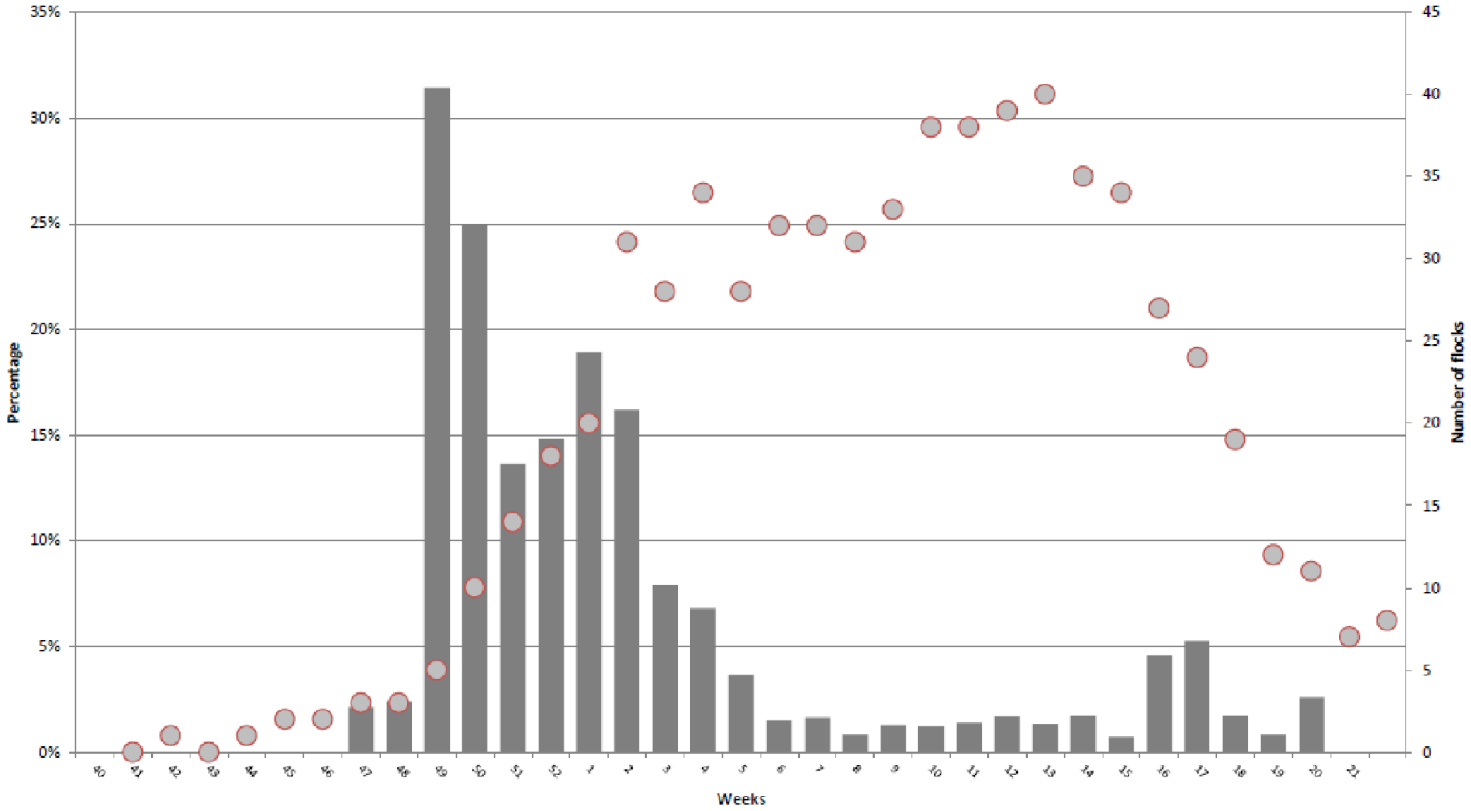
Percentage of malformed lambs born per week out of total number of lambs born per week (bars), and the number of flocks with sheep lambing (dots) in the period from October 3^th^ 2011 to May 27^th^ 2012, based on the questionnaire.

Based on the results of the questionnaires, the majority of malformed lambs in 80 case flocks was between the end of November 2011 (week 49) and the end of January 2012 (Figure 3).

Most frequently observed SBV-related clinical signs by case flocks owners were a posterior presentation (33.3%), prolonged parturition (50.0%), dystocia (45.6%), neurological symptoms in lambs (37.8%), abnormal abdominal or uterine fluid of the ewe (23.3%), and lambs with hydrops ascites (23.3%) (Table 2).

**Table 2:** Flock owner reported clinical signs in 90 sheep flocks with malformed lambs in the lambing season 2011/2012 in the Netherlands.

Group	Symptom	Percentage of flocks
Behaviour of ewe	Ewe slow	17.8%
	Ewe spends more time lying	12.2%
Parturition	Abnormal uterine fluid	23.3%
	Caesarean section	8.9%
	Dystocia	45.6%
	Posterior presentation	33.3%
	Prolonged parturition	50.0%
Lambs	Hydrops ascites lambs	23.3%
	Neurological symptoms lambs	37.8%
Udder health	Abnormal udder development	15.6%
	Abnormal consistence of the milk	4.4%
	Mastitis	10.0%
	Insufficient amount of milk	18.9%

A significant difference in abnormal suckling behavior in lambs was seen between the 88 case and 65 control flocks (*p-*value*<0.01;* Pearson’s chi-squared test). Twenty-nine (33.0%) of the case flocks and four (6.2%) control flocks reported this symptom. Clinical symptoms in ewes before, during or after the mating period was reported by participating flock owners in 10.0% of 90 case flocks and in 6.1% of 82 control flocks, which was not significantly different. Diarrhoea before, during or after the mating period was not significantly different and reported in 16.7% of 90 case and 20.7% of 82 control flocks.

### Effects of SBV Infection on Reproductive Performance and Mortality Rates

Based on questionnaire information, no significant differences were found of the effect of SBV on reproductive performance and mortality rates between case and control flocks, except regarding repeat breeders (Table 3). From 70 case flocks, 4.3%, 52.9% and 42.9% observed less, comparable or more repeat breeders, respectively, in the lambing season of 2011/2012 compared to the previous season. From 69 control flocks, 15.9%, 63.8% and 20.3% observed less, comparable or more repeat breeders in the breeding season of 2011/2012, respectively, compared to the previous season, which was significantly different (*p<0.01*).

**Table 3:** Reproductive performance and mortality rates in 90 case and 82 control flocks in the lambing season of 2011/2012 compared to the previous lambing season, as reported by participating flock owners.

Variable	Group	N	Less	Comparable	More	*P*-value^1^
Abortion	Case	69	13.0%	79.7%	7.2%	0.73
	Control	69	11.6%	84.1%	4.3%	
Barren ewes	Case	76	15.8%	50.0%	34.2%	0.84
	Control	77	19.5%	48.1%	32.5%	
Lambing rate	Case	71	32.4%	54.9%	12.7%	0.16
	Control	72	20.8%	56.9%	22.2%	
Mortality rate lambs	Case	86	5.8%	64.0%	30.2%	0.21
	Control	81	7.4%	74.1%	18.5%	
Mortality rate rams	Case	83	3.6%	91.6%	4.8%	0.94
	Control	75	2.7%	92.0%	5.3%	
Mortality rate ewes	Case	89	9.0%	71.9%	19.1%	0.75
	Control	81	8.6%	76.5%	14.8%	
Number of lambs per ewe	Case	79	29.1%	46.8%	24.1%	0.50
	Control	80	21.3%	53.8%	25.0%	
Repeat breeders	Case	70	4.3%	52.9%	42.9%	<0.01
	Control	69	15.9%	63.8%	20.3%	

1 *p-*value following Pearson’s chi-squared test.

The number of lambs per ewe was 1.69 in 45 case flocks, and 1.74 in 58 control flocks in the lambing period 2011/2012, and not significantly different. In 45 case flocks, 13.9% of the lambs was born dead or died before weaning compared to 8.3% in 58 control flocks (*p-*value*<0.01*; *t-*test). The percentage of malformed lambs was higher in 45 case flocks (5.2%) than in 58 control flocks (0.3%) (*p-*value*<0.01*; Pearson’s chi-squared test).

### Risk Factors for Malformations in Newborn Lambs

Variables that were included in the full model however have been excluded during the model selection procedure were total number of sheep, vaccination with Heptavac, scrapie resistant accredited, synchronization, deworming, introduction of ewe(s), introduction of ram(s), sold ewe(s), sold ram(s). Model fit statistics of the full model (Pseudo R2 of 0.3751/AIC of 166.4) were slightly better than the final model, yet log likelihood ratio testing indicated a non-significant improvement (p = 0.885). In the final multivariable model on flock-level malformations in newborn lambs, based on 169 observations, the factors ‘start of mating season’, ‘purchase of silage’, and ‘presence of one of more dogs’, remained in the model after the backwards selection procedure. Malformations in newborn lambs were more likely in flocks in which the flock owner had one or more dogs (OR = 3.3; 95% CI: 1.3–8.3; *p-*value = 0.01), in flocks that purchased silage (OR = 5.0; 95% CI: 1.7–15.0; *p-*value<0.01), and in flocks in which the start of the mating season was before August 2011 (OR = 33.1; 95% CI: 10.0–109.8; *p-*value<0.01) or in August 2011 (OR = 8.2; 95% CI: 2.7–24.6; *p-*value<0.01) compared to October 2011 as reference period. No difference was found between the reference period (October 2011) and September 2011 (OR = 1.3; 95% CI: 0.5–3.8; *p-*value = 0.60), and November-December 2011 (OR = 0.4; 95% CI: 0.0–4.5; *p-*value = 0.48).

## Discussion

This study aimed at identifying flock-level effects of SBV infection on reproductive performance and mortality rates, as well as at identifying and quantifying risk factors for malformations. SBV has rapidly emerged throughout the Netherlands in the second half of 2011, infecting a large proportion of the ruminant population [6]. During the epidemic of 2011 and 2012, it became clear that the extent of the within-flock impact was diverse, varying from no clinical expression of the disease, to severe congenital malformations in varying percentages of newborn lambs and loss of ewes caused by dystocia [4]. We knew in an early stage of the outbreak that SBV probably had been disseminated all over the country, and we expected that a high proportion of all sheep and sheep flocks had been exposed. We did not know the risk factors for malformations and impact on reproductive performance and mortality rates, and think that this study has revealed this knowledge gaps. Exposure of both case and control flocks to SBV did not influence the aim of our study. In this study, a case-control study design was chosen, based on absence or presence of malformations in newborn lambs. During the study period, owners of control flocks reported 0.3% lambs with malformations, however they did not report the specific congenital malformations seen in lambs submitted for post mortem examination in suspicion of SBV. In Australia, under normal circumstances, an incidence of 0.2–2.0% of malformations in newborn lambs has been reported [14]. The GD carries out monitoring of animal health in the Netherlands, and congenital malformations in lambs were scarce until December 2011: between January 2006 and July 2011, only eight out of 4,787 (0.17%) lambs submitted for post mortem examination, ten out of 7,873 (0.13%) telephone help desk questions asked on small ruminants, and four out of 2,042 (0.2%) flock visits carried out by small ruminant health specialists of the GD were related to congenital malformations (unpublished data). Therefore, the case-control classification of the flocks seems to be valid.

### Seroprevalence

In control flocks, serum samples were collected with a slight time delay compared to sampling in case flocks, as the selection of control flocks depended on voluntary application of flock owners and absence of SBV related malformed lambs in the lambing season. Samples from case flocks were collected from January 5^th^ 2012 until October 30^th^ 2012, and from control flocks between July 3^th^ 2012 and October 29^th^ 2012. A possible SBV transmission in 2012 could have caused seroconversion in naïve animals mainly towards the end of our study period. To examine this, animal level seroprevalences were re-estimated, these seroprevalences were not different from seroprevalences during the whole study period. No significant difference in seroprevalence was found between case and control flocks. This is in line with a serosurvey on SBV in sheep in France [5]. Because of the relatively low number of flocks that submitted sufficient samples for a reliable within-flock seroprevalence estimation, it was decided to estimate within-flock seroprevalence based on flocks that submitted 50 or more samples. As a consequence, power and confidence were relatively low. Nevertheless, our seroprevalence estimates seem to be comparable to what is described in Belgium, France and the Netherlands in areas with reported outbreaks of congenital malformations due to SBV [5], [6], [15]. Estimated seroprevalences in ewes that gave birth to clinically normal lambs (75.7%), did not differ significantly from seroprevalence in ewes that gave birth to one or more malformed lambs (88.3%). Why this latter percentage is not 100% is unclear. Misclassification of ewes with malformed lambs, malformations in lambs not caused by SBV, or false negative ELISA results might have played a role, but it cannot be explained with certainty form the results of this study.

### Effects of SBV Infection

In malformed lambs submitted for this study for post mortem investigation during the first months after the start of this epizootic outbreak, approximately 35% of brain tissue samples were positive for SBV. Virus detection techniques from neonatal foetuses such as virus isolation, RT-qPCR and immunohistochemistry are not always successful, probably leading to underdiagnoses [2], [16], [17], most likely due to the lag between time of infection and time of sampling combined with the occurrence of a short viraemic period and clearing of virus by foetal neutralizing antibodies [9].

The percentage of ewes with disease before, during or after the mating period, including diarrhoea which was considered a clinical sign in dairy cattle [1], was not significantly different between case and control flocks. This was an expected result, because of the high seroprevalences in both case and control flocks. In many flocks that reported diarrhoea, other causes, for example dietary changes and infection with gastrointestinal nematodes, could not be excluded. Experimental infection of sheep in Germany confirm these results, as only one out of thirty sheep showed diarrhoea shortly after inoculation with SBV [9].

An increased number of barren ewes was reported in both case and control flocks compared to the previous lambing season. In case flocks, significantly more repeat breeders were observed. Early embryonic death might explain the higher proportion of barren ewes and the higher percentage of repeat breeders in case flocks. Early embryonic death is mainly associated with teratogenic influences during the first 30 days of gestation [18]. So, repeat breeding was possibly a proxy for SBV infection early gestation or earlier in the year 2011. Another explanation for repeat breeders could be a raised body temperature in SBV infected rams, which influences semen quality [19]. Abnormal suckling behaviour, probably due to malformations in the brain, was significant different between case and control flocks, although exposure to SBV was more or less equal. In general, presentation of congenital malformations is not only dependent on the teratogenic agent itself, but also on the pregnancy stage at which the dam came into contact with the teratogenic agent [20]. Ovine congenital malformations like arthrogryposis, torticollis, scoliosis, kyphosis and malformations of the brain and spinal cord have been described after contact with a teratogenic agent roughly between day 30 and day 60 of gestation [18], [21]–[23]. So, abnormal suckling behaviour may be a confounder for (non-lethal) malformation. Due to recall bias, it may be that flock owners that have observed malformations in lambs are more likely to incorrectly state the presence of clinical signs in ewes and lambs, than those that did not observe malformations. Also, administration of data by sheep flock owners was often incomplete, so accurate numbers were often not available.

### Risk Factors

In this study, as expected, a delayed start of the mating period decreased the odds of malformations in newborn lambs. Start of mating season was defined as the month in 2011 in which the ram was first introduced to the sheep flock. In case flocks, the highest odd of malformed lambs was in sheep mated before and in August 2011. In the Netherlands, only a small number of flocks had a lambing period between July and November 2011, and no congenital malformations were reported in this period. So highest risk on malformed lambs was in ewes mated in July or August 2011. This supports the results of a study in the Netherlands, that showed that introduction of SBV was most likely in August 2011 ([6]. Besides, Culicoides biting midges are most likely vectors for SBV ([24], so risk of infection will also highly depend on their seasonal activity.

The average flock size in case and control flocks was different, which could have influenced our model estimates. Therefore, the multivariable model was fitted again without flocks with a flock size of more than 600 animals (n = 14). As associations were only slightly altered, flock size seemed to have a negligible influence on the risk factors that were found.

In flocks with one or more dogs, an increased odd of malformations in newborn lambs was found. Recently, SBV-specific antibodies were found in a dog in Sweden [25] and RT-qPCR SBV positive results were found in a dog in France [26]. A possible link between SBV infections and dogs needs further investigation. An explanation could be that dogs get orally infected with SBV, like it has been described for bluetongue virus [27]. Although, presence of dogs could also be a proxy for underlying differences in management. On farms that purchased silage, an increased odd of malformations in newborn lambs was found. Purchase of silage could also be a proxy for underlying differences in management, for example the period that sheep are kept indoors, which was not questioned in this study. So it is unknown if purchase of silage has been, from a biological point of view, a real risk factor, so far, no evidence is found in other studies.

## Conclusions

SBV infection resulted in congenital malformations in newborn lambs, and a rise in the percentage of stillborn lambs and lambs that died before weaning, lambs with abnormal suckling behaviour, and repeat breeders. Our study indicated associations between malformations in lambs and the start of the mating period, presence of dogs and purchase of silage. In adult sheep, clinical signs after SBV infection seem to be limited, although some farmers reported loss of ewes as a result of dystocia. High seroprevalences were found, which were not significantly different between case and control flocks. Because of a combination of high seroprevalences and a presumed long lasting immunity after infection with SBV, risk associated with possible future SBV infection is mainly expected in early mated seronegative sheep. Besides vaccination, delay of the mating period at least until vector-activity is decreasing, could be a potential preventive measure for naïve animals to reduce SBV induced losses, depending on the survival and persistence of the virus in Northwestern Europe.
